# Case Report: Alpelisib-Induced Drug Reaction With Eosinophilia and Systemic Symptoms: A Rare Manifestation of a Common Side Effect

**DOI:** 10.3389/fonc.2021.726785

**Published:** 2021-08-24

**Authors:** Umair Majeed, Tudor Puiu, Jason Sluzevich, Gina Reynolds, Marites Acampora, Alvaro Moreno-Aspitia, Katherine J. Bodiford, Pooja Advani

**Affiliations:** ^1^Division of Hematology Oncology, Mayo Clinic Florida, Jacksonville, FL, United States; ^2^Division of Dermatology, Mayo Clinic Florida, Jacksonville, FL, United States

**Keywords:** breast cancer, DRESS, drug rash, PI3K inhibitor therapy, alpelisib

## Abstract

Alpelisib is a PIK3a inhibitor approved for the treatment of metastatic ER+ breast cancer in combination with fulvestrant. Although rash is a common side effect of this medication, we present the first case of drug reaction with eosinophilia and systemic symptoms (DRESS) upon initial exposure to alpelisib. Here we describe the clinical-pathological findings and management of our patient with alpelisib-induced life-threatening DRESS syndrome. The goal of this case report is to highlight association of alpelisib with DRESS syndrome, in clinical practice, so that alpelisib can be immediately stopped and treatment for this serious condition promptly initiated.

## Introduction

Drug reaction with eosinophilia and systemic symptoms (DRESS) is a severe adverse drug reaction characterized by an extensive skin rash in association with visceral organ involvement, lymphadenopathy, eosinophilia, and atypical lymphocytosis. It is most commonly associated with antiepileptic medications (1). Metastatic breast cancer is one of the leading causes of cancer-related deaths in the United States; almost 170,000 women are estimated to be living with this disease in 2020 (2). Activating PIK3CA mutations occur in approximately 40% of patients with hormone receptor (HR)-positive, human epidermal growth factor receptor 2 (HER2)-negative breast cancer (3). Alpelisib is an alpha specific PI3K inhibitor that selectively inhibits P110α (4). In May 2019, alpelisib in combination with fulvestrant was approved for HR positive, HER2 negative, advanced breast cancer in patients with disease progression on endocrine therapy (5). Rash was the second most common side effect amongst the reported grade 3 or higher adverse events (6). Alpelisib-induced DRESS is a rare but life-threatening side effect with only one case reported in the literature of a patient who presented with this complication within 24 h of being re-challenged with a reduced dose of alpelisib after she had already cleared the initial rash (7). Here we present the first case of alpelisib-induced DRESS syndrome as an early and initial manifestation of dermatologic adverse event associated with this targeted agent.

## Case Report

**Figure 1:**
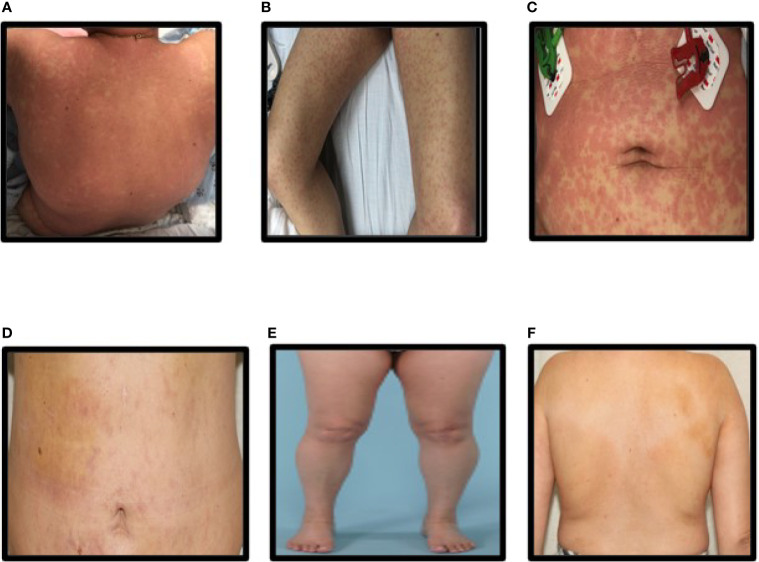
**(A–C)** Rash on the back, legs, and abdomen at presentation, **(D–F)** Rash on the back, legs, and abdomen after steroid treatment.

A 52-year-old female with metastatic HR positive (ER+ and PR+), HER2 negative breast cancer involving liver, bone, and pleural cavity presented to the hospital with fever of 102°F, chills, and non-pruritic generalized body rash of 1 day duration. She had initiated treatment with alpelisib (300 mg oral daily) plus fulvestrant (500 mg intramuscular injection) and cetirizine prophylaxis 12 days prior to presentation. Patient did not receive any CYP3A4 inhibitors. Rash started on her trunk (**Figures 1A, C**) and rapidly progressed to involve her upper and lower extremities (**Figure 1B**). She was also noted to have a facial rash with periorbital swelling. There was no involvement of mouth, genital area, or eyes. On physical examination, deep red macules and papules coalescing into patches and thin plaques were seen in the extremities and trunk (**Figures 1A–C**).

**Figure 2:**
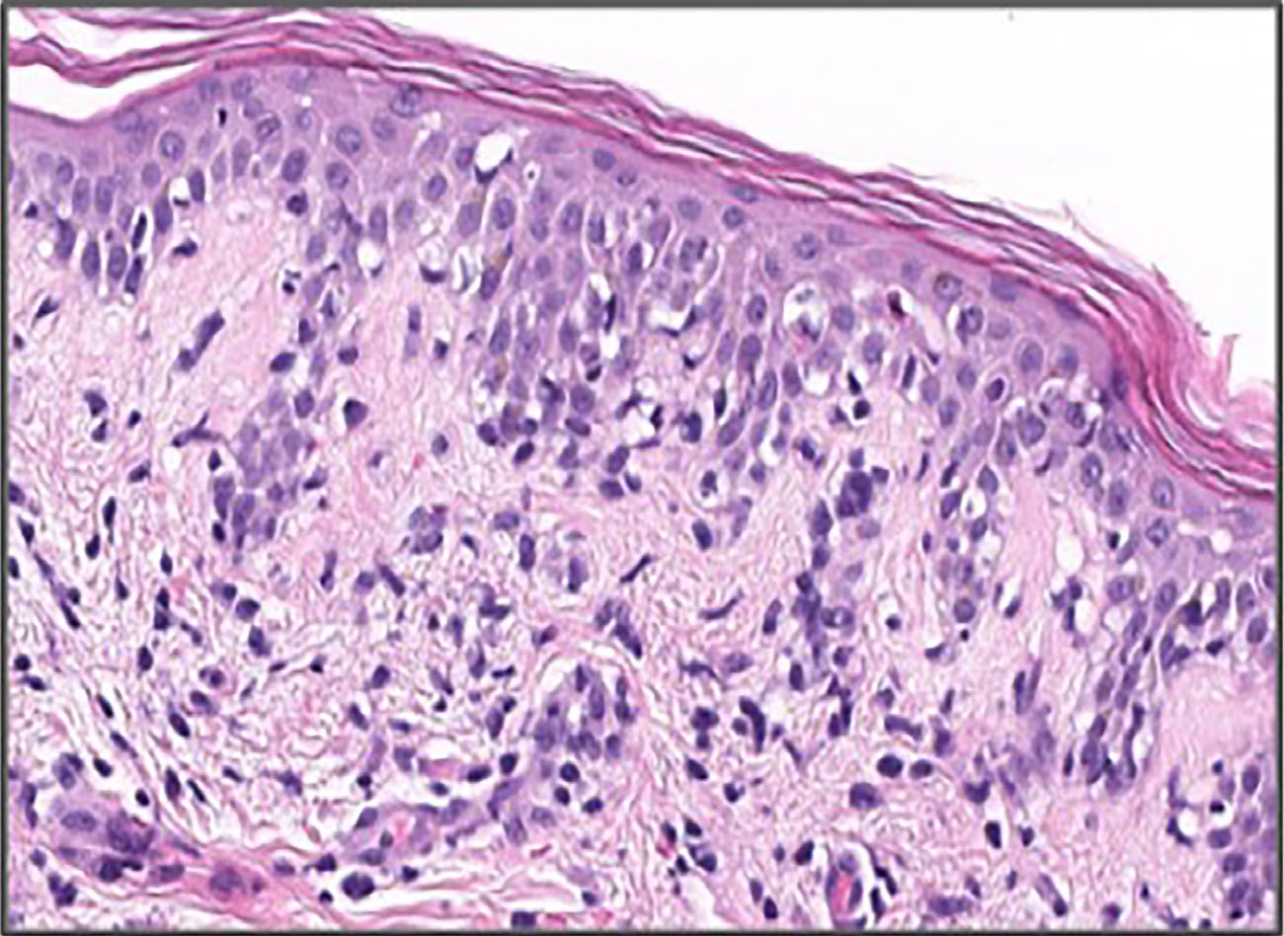
H and E stain of right lower abdomen punch biopsy showing focal epidermal dyskeratosis, mild spongiosis, and moderate vacuolar interface dermatitis in association with superficial perivascular lymphohistocytic inflammation.

Complete blood count was remarkable for leukocytosis (12,700) with elevated absolute eosinophil count of 1,310 and new onset thrombocytopenia of 72,000. Her AST, ALT, and bilirubin were elevated to 74 U/L, 46 U/L, and 1.7 mg/dl, respectively, from a normal baseline 4 days earlier. Left lower abdomen skin punch biopsy showed spongiotic dermatitis with focal interface dermatitis and high epidermal cytoids characteristic of morbilliform drug eruption seen in the setting of DRESS syndrome (**Figure 2**).

Based on the RegiSCAR criteria (**Supplementary Table 1**) and RegiSCAR scoring system (**Supplementary Table 2**), this patient met criteria for DRESS (score >5) with fever, eosinophilia, presence of atypical lymphocytes, thrombocytopenia, elevated liver enzymes, skin rash involving >50% of body surface area with appearance and biopsy findings suggestive of DRESS, along with absence of other identifiable causes.

Alpelisib was immediately discontinued, and patient was started on treatment with oral prednisone 1 mg/kg. Patient was discharged on hospital day number 3 after improvement in her rash. Follow-up at 1 week showed significant improvement in the rash (**Figure 1D**), normalization of eosinophilia, and improvement in thrombocytopenia and elevated liver enzymes. Patient was continued on oral prednisone 1 mg/kg for 14 days and then completed a slow taper of prednisone over 12 weeks with complete resolution of thrombocytopenia and elevated liver enzymes. **Figures 1E, F** show complete resolution of rash after 2 weeks of prednisone therapy.

## Discussion

DRESS is a rare, life-threatening drug-induced reaction occurring in 0.9 to 2 per 100,000 patients per year (8). A clear drug trigger can be identified in about 80% of the cases with majority of cases attributed to high-risk drugs such as anticonvulsants (carbamazepine, phenytoin, lamotrigine), allopurinol, and sulfonamide-containing antibiotics (1). It is hypothesized that DRESS is a T-cell-mediated hypersensitivity reaction with expansion of circulating activated CD4+ and CD8+ T lymphocytes. Reactivation of viruses from the Herpesviridae family (HHV6, HHV7, EBV, and CMV) can occur in up to 75% of these patients (9). Alpelisib is a PI3K inhibitor, and the PI3K/AKT pathway has been demonstrated to have an important role in keratinocyte differentiation. The inhibition of this pathway blocks the expression of certain growth and differentiation markers, thus leading to epidermal cell death including apoptosis of keratinocytes (10). The combination of alpelisib with fulvestrant was studied in the phase III SOLAR-1 clinical trial, which demonstrated significant improvement in progression-free survival of 11.0 months *vs* 5.7 months in the placebo-fulvestrant group, in patients with PIK3CA-mutated metastatic breast cancer (6). Rash (all grade) was seen in 53.9% of patients in the combination alpelisib with fulvestrant group. It was the second most frequent (20.1%) grade 3 or 4 Adverse Event (AE) leading to discontinuation of alpelisib in 3.2% of patients. A retrospective review of four randomized clinical trials, studying alpelisib in metastatic breast cancer, evaluated 102 patients with alpelisib-related dermatologic AE (7). In this analysis, only one case of DRESS was reported in the context of alpelisib rechallenge in a patient who previously stopped the drug due to a rash (7), which is different from our patient who developed the rash on first exposure to alpelisib. DRESS syndrome usually manifests 2–3 weeks after the introduction of the culprit drug as a maculopapular morbilliform exanthema and fever (9), similar to our patient in whom the latent period was 12 days. The cutaneous lesions can become confluent after starting as erythematous patchy macules with symmetrical distribution on the trunk and extremities. The most characteristic cutaneous lesions during the early phase are periorbital and facial edema that are present in 70% of the cases, while the mucosal areas are usually spared (11). Our patient had the classic laboratory findings of DRESS syndrome including eosinophilia, which is present in >95% of the patients; lymphocytosis with atypical lymphocytes; and elevation in liver enzymes, which is seen in up to 70% of the patients (1). As in 75% of the patients with DRESS who have reactivation of Herpesviridae family member, EBV PCR viral load was detectable in our patient. Histopathology can show interface dermatitis with basal vacuolization and features of vascular damage in up to 75% of the patients (12). Diagnosis of DRESS can be made based on diagnostic criteria established by using the RegiSCAR criteria and scoring system (**Supplementary Tables 1** and **2**) (1). Differential diagnosis can be broad, such as acute cutaneous lupus erythematosus, viral infections (CMV, measles, HIV, viral hepatitis), cutaneous T lymphoma can be associated with a generalized rash; other drug-related cutaneous reactions such as exanthematous drug eruptions, acute generalized exanthematous pustulosis (AGEP), and Stevens-Johnson syndrome should also be kept in mind. Hypereosinophilic syndromes can have cutaneous manifestation in addition to eosinophilia (9). Prognosis for DRESS can be variable and unpredictable as it can be complicated by myocarditis, *Pneumocystis jirovecii* pneumonia, sepsis, and gastrointestinal bleeding leading to significant morbidity and mortality (9). Hence, prompt recognition of DRESS syndrome is crucial. Shiohara et al. have proposed a composite score to assess severity and predict outcomes of DRESS patients by stratifying these patients into three groups; mild (score <1), moderate (score 1–3), and severe (score ≤4), based on early or late scores to predict the risk of complications (**Supplementary Table 3**) (9).

Management usually involves identification and withdrawal of the causative medication. Emollients, high-potency topical steroids, and antihistamines can help control pruritus. For mild disease with no organ involvement, withdrawal of the causative drug and symptom control may be all that is needed (13). In patients with single or multiple organ involvement, systemic glucocorticoids are used as first-line therapy. Prednisone can be used at 1 mg/kg tapered over 8–12 weeks (14). Second-line therapies such as cyclosporine (15), IVIG (16), other immunosuppressive agents such as tofacitinib (17), and cyclophosphamide (18) can be considered if patients do not response to first-line therapy with glucocorticoids. Use of antiviral therapy is not recommended as viral reactivation usually resolves spontaneously. Mortality rate is estimated to be around 2–10% with most patients recovering within weeks to months after drug withdrawal (1).

## Conclusion

In summary, rash is a common adverse effect related to alpelisib; however, rarely patients can develop the rare syndrome of DRESS, which requires prompt recognition due to associated morbidity and mortality. Clinical presentation and laboratory evaluation using the RegiSCAR criteria can help diagnose this condition. Alpelisib should be immediately stopped in all cases, and treatment with high-dose steroids should be initiated for patients with moderate to severe syndrome, until resolution of symptoms and lab abnormalities. Steroids should then be gradually tapered as relapse or flareups can occur days to weeks after stopping the offending agent.

## Data Availability Statement

The original contributions presented in the study are included in the article/**Supplementary Material**. Further inquiries can be directed to the corresponding author.

## Ethics Statement

Written informed consent was obtained from the individual(s) for the publication of any potentially identifiable images or data included in this article.

## Author Contributions

UM and PA wrote the manuscript. TP, JS, GR, MA, AM-A, and KB reviewed and edited the manuscript. JS also helped with pathology slide and explanation. All authors contributed to the article and approved the submitted version.

## Conflict of Interest

The authors declare that the research was conducted in the absence of any commercial or financial relationships that could be construed as a potential conflict of interest.

## Publisher’s Note

All claims expressed in this article are solely those of the authors and do not necessarily represent those of their affiliated organizations, or those of the publisher, the editors and the reviewers. Any product that may be evaluated in this article, or claim that may be made by its manufacturer, is not guaranteed or endorsed by the publisher.

## Supplementary Material

Supplementary Table 1 Registry of severe cutaneous adverse reaction criteria for diagnosis of drug rash and eosinophilia with systemic symptoms (RegiSCAR criteria) (1). *Necessary criteria, ǂ Three out of four criteria required.

Supplementary Table 2 Registry of severe cutaneous adverse reaction diagnosis score for drug rash and eosinophilia with systemic symptoms (RegiSCAR scoring system) (1). Total score: 8 (<2, no DRESS syndrome; 2–3, possible DRESS; 4–5, probably DRESS; ≥6, definite DRESS) Registry of Severe Cutaneous Adverse Reactions.

Supplementary Table 3 Composite scores for evaluating the severity of drug-induced hypersensitivity syndrome and drug reaction with eosinophilia and systemic symptoms (DRESS) and predicting the disease outcomes (9).
