# Supplementary material for: Case Report: Alpelisib-Induced Drug Reaction With Eosinophilia and Systemic Symptoms: A Rare Manifestation of a Common Side Effect
Source: Front Oncol. 2021 Aug 24;11:726785. doi: 10.3389/fonc.2021.726785 (PMC8421674; doi:10.3389/fonc.2021.726785)
Supplement: Supplementary Table 1 — Registry of severe cutaneous adverse reaction criteria for diagnosis of drug rash and eosinophilia with systemic symptoms (RegiSCAR criteria) (1). *Necessary criteria, ǂ Three out of four criteria required. [file Table_1.docx]

**Supplementary Material**

**Supplementary Table 1:** Registry of severe cutaneous adverse reaction criteria for diagnosis of drug rash and eosinophilia with systemic symptoms (RegiSCAR criteria) [1]

| 1 | Hospitalization* |
| --- | --- |
| 2 | Reaction suspected to be drug related* |
| 3 | Acute Rash* |
| 4 | Fever>38$^{\circ}C$^ǂ^ |
| 5 | Enlarged lymph nodes at a minimum of 2 sites ^ǂ^ |
| 6 | Involvement of at least 1 internal organ ^ǂ^ |
| 7 | Blood count abnormalities (Lymphocytosis, eosinophilia, thrombocytopenia) ^ǂ^ |

Necessary criteria

ǂ Three out of four criteria required

**Supplementary Table 2:** Registry of severe cutaneous adverse reaction diagnosis score for drug rash and eosinophilia with systemic symptoms (RegiSCAR scoring system) [1]

| Features | No | | Yes | Unknown |
| --- | --- | --- | --- | --- |
| A. Fever>38$.5^{\circ}C$ | -1 | | 0 | -1 |
| B. Enlarged lymph nodes (> 2 sites, >1 cm) | 0 | | 1 | 0 |
| C. Atypical Lymphocytes | 0 | | 1 | 0 |
| D. Eosinophilia   1. 700-1499 2. ≥1500 | 0 | | 1 | 0 |
|  |  | | 2 |  |
| E. Skin Rash   1. Extent>50 2. At least 2: Edema, infiltration, purpura, scaling 3. Biopsy suggesting DRESS | 0 | 1 | |  |
|  | -1 | | 1 |  |
|  | -1 | | 0 |  |
| E. Internal organ involvement   1. One 2. Two | 0 | | 1 | 0 |
|  |  | | 2 |  |
| F. Resolution in ≥15 days | -1 | | 0 | -1 |
| G. Ruling out other causes   1. (>3) of ANA, blood culture, serology for HAV/HBC/HCV, Chlamydia/mycoplasma | 0 | | 1 | 0 |

Total score: 8 (<2 no DRESS syndrome, 2-3 possible DRESS, 4-5 Probably DRESS, ≥6 definite DRESS) Registry of Severe Cutaneous Adverse Reactions

**Supplementary table 3:** Composite scores for evaluating the severity of drug-induced hypersensitivity syndrome and drug reaction with eosinophilia and systemic symptoms (DRESS) and predicting the disease outcomes [9]

| **Score** | -1 | 1 | 2 | 3 |
| --- | --- | --- | --- | --- |
| **Fixed parameters** |  |  |  |  |
| Age (yr) | ≤40 |  | ≥75 |  |
| Duration of drug exposure after onset |  | ≥7 days |  |  |
| Allopurinol exposure |  | Yes |  |  |
| **Variable parameters** |  |  |  |  |
| Prednisolone intake |  |  | Pulse |  |
| Skin involvement |  |  |  |  |
| Erythematous rashes (% body surface area) |  | ≥70% | Erythroderma |  |
| Erosive lesions (% body surface area) |  | 10–29% |  | ≥30% |
| Fever ≥ 38.5 °C (duration) |  | 2–6 days | ≥7 days |  |
| Appetite loss |  | ≥5 days with ≤70% of regular food intake |  |  |
| Renal dysfunction (creatinine, mg/dl) |  | 1.0–2.0 |  | ≥2.1 or HD |
| Liver dysfunction (ALT, IU/l) |  | 400–1000 | ≥1001 |  |
| C-reactive protein (mg/dl) | ≤2 | 10–15 | ≥15.1 |  |
